# The management success of the invasive late goldenrod (*Solidago gigantea* Aiton.) in a nature conservation area is strongly related to site, control measures and environmental factors

**DOI:** 10.7717/peerj.13161

**Published:** 2022-04-19

**Authors:** Rea Maria Hall, Bernhard Urban, Hans-Peter Kaul

**Affiliations:** Institute of Agronomy, University of Natural Resources and Life Science Vienna, Tulln an der Donau, Austria

**Keywords:** Late goldenrod, Invasion in national parks, Management intensitiy, Biodiversity loss due to invasion, Mowing regimes, Rotary tillage, Land use change

## Abstract

The late goldenrod (*Soldiago gigantea* Aiton; Asteraceae) is one of the most abundant invasive species in various types of habitats. Its long-creeping plagiotropic rhizomes enable the plant to build up dense, monospecific stands within a short time. Particularly in nature conservation areas, the invasion of goldenrod can cause severe disruptions in the naturally occuring mutualims between plants, insects and higher trophic levels, subsequently impeding the achievement of nature conservation goals. As management options of goldenrod in nature conservation areas are limited, this three-year study aimed to test the effectiveness of three management treatments (two-time mowing, triticale cultivation, and reverse rotary cutting) on four different sites in the Austrian Donau-Auen National Park. The number and height of goldenrod shoots were recorded three times a year on twelve permanent trial plots on each site to test for the effectiveness of the treatments. In addition, vegetation surveys were performed to observe the recovery potential of native plant species. Even though the three-years mowing and the triticale cultivation reduced goldenrod by 95.6% and 97.2% resp., we could find no relation between the effectiveness of the treatment and the intensity of disturbance created by the control option. On the contrary, with a reduction of only 5.4% in goldenrod density the most intensive treatment, the rotary cutting, showed the lowest efficiency. The highest positive effect on the re-establishment of native plant species was recorded with two mowing events per year. Even though the study revealed that certain management options have the potential to effectively reduce goldenrod and to simultaneously increase the establishment success of native species, results can only be seen as so-called snapshots. For example, as shown on site EJW one unforeseeable wild boar digging event transformed a 84.5% reduction into a 4.7% increase in goldenrod density. Therefore, a proper and regular monitoring is essential to be able to react to the effects of unpredictable events that can have severe impact on vegetation dynamics.

## Introduction

Climate change, land use change (agricultural intensification *vs.* land degradation or dereliction), and increased human travel and trading are the main drivers promoting deliberate or inadvertent introductions of alien invasive species (Vitousek et al., 1997; Buckland et al., 2001; Standish, Cramer & Hobbs, 2008). Recently, it was shown that the introduction, establishment and distribution of invasive species is nowadays the second most important cause of species extinction globally after habitat destruction (Fitoussi, Pen-Mouratov & Steinberger, 2016; Čerevková et al., 2019). One of the most troublesome invaders in many European countries is the late goldenrod (*Solidago gigantea* Aiton), a rhizomatous perennial herb, native to North America (Weber & Jakobs, 2004; Vanderhoeven et al., 2006). The late goldenrod (subsequently called goldenrod) was introduced in Europe in the 18th century as an ornamental and melliferous plant. It started to escape from ornamentals and spread extensively in the decades between 1850 and 1880 (Weber & Jakobs, 2004). Due to its plasticity to respond to changes in environmental conditions by adjusting its growth patterns the plant was able to invade in various types of habitats, *i.e.,* ruderal areas, roadsides, embankments, riverside habitats (Ellenberg et al., 2001; Jakobs, 2004; Weber & Jakobs, 2004). Particularly in the last decades, social and economic changes have driven the abandonment or at least a reduced, more sustainable usage of former farmland to increase biodiversity and conservation value of agricultural areas and to provide ecosystem services like pollination (Tscharntke, Batáry & Dormann, 2011; Fenesi et al., 2015). However, these old fields and fallow land provided ideal habitats for invasive species in general and goldenrod in particular as the plant has a very high colonization ability (Standish, Cramer & Hobbs, 2008; Fenesi et al., 2015). Single plants grow rapidly up to 2 m height and can produce up to 10,000 light, wind-dispersed seeds which long-distance distribution is facilitated by a pappus (Kabuce & Priede, 2010; Kajzer-Bonk, Szpilyk & Woyciechowski, 2016). Moreover, the plant produces long-creeping plagiotropic rhizomes (up to 50 rhizomes per ramet) that enables the plant to build up dense, monospecific stands within a very short time (Weber & Jakobs, 2004). Consequently, natural succession pathways and patterns of vegetation recovery especially on old fields and fallow land are altered by preventing the establishment of native plant species (Cramer, Hobbs & Standish, 2008; Fenesi et al., 2015). Studies showed that areas invaded by alien goldenrods show significantly lower plant diversity by outcompeting natural communities but also by changing local biotic factors like soil properties (*i.e.,* increased acidity, changes in soil moisture) and biogeochemical cycles through dead biomass accumulation (Chapuis-Lardy et al., 2006; Baranová, Manko & Jászay, 2014; Čerevková et al., 2019). By reducing floral diversity, monospecific stands of goldenrods have detrimental effects on insect populations (*i.e.,* pollinators) and subsequently on taxa from higher trophic levels such as birds causing severe disruptions in naturally occurring mutualism (Moroń et al., 2009; Skórka, Lenda & Tryjanowski, 2010).

Particularly in protected areas, these shifts in community structures due to alien invasion are a major concern of ecologist and natural conservationists. Even though numerous studies implied that these natural ecosystems are more resistant to invasion by alien species due to a high abundance of native species with a high degree of naturalness, most national parks are not isolated from surrounding landscapes. Rather they exist in a matrix of intensive human usage, and many types of human activity that could disturb the ecological resilience, enabling the invasion of alien species into protected areas (Pauchard & Alaback, 2004; Braun, Schindler & Essl, 2016; Foxcroft et al., 2017).

This is also true for the Austrian Donau-Auen National Park, which is part of the Marchfeld Plain, one of the most intensively used agricultural areas in Austria. Additionally, due to its location next to the border of Austria’s capital Vienna, the Donau-Auen National Park is a popular destination for thousands of cyclers, hikers and nature lovers throughout the year.

Until its foundation in 1996, also the area of the national park was under agriculture use and until today agriculture and particularly grassland management plays a vital role in the achievement of nature conservation goals. The Donau-Auen National Park comprises nine habitat types listed in the Fauna-Flora-Habitat Directive (92/43/EWG), including semi-natural dry grassland (habitat type 6210 Annex 1), sub-pannonic steppic grassland (habitat type 6240 Annex 1) and lowland hay meadows (habitat type 6510 Annex 1). To preserve these habitat types, protected meadows management concepts (*i.e.,* mowing regimes) were developed to prevent these meadows from natural succession like scrub encroachment (Donau-Auen National Park, 2019). However, due to changes in legal regulations (*i.e.,* the Austrian Agri-Environmental Programme “ÖPUL”), especially in the last 15 years, a couple of trade-offs in conservation and management goals evolved. For example, to ensure that ground-breeding birds can complete their breeding season without disturbance, mowing was not allowed before mid of August which indirectly promoted the establishment of goldenrod: After germination or sprouting goldenrod builds up biomass rapidly by intensive shoot elongation and leaf formation, leading also to the production of rhizomes within four weeks (Weber & Jakobs, 2004). To weaken the plant, it would be necessary to remove leaf area through an early cutting (plant height around 30 cm), in order to reduce the photosynthetic potential, leading to lower assimilation rates and thus, to a lower allocation of assimilates to the rhizome. In addition, for resprouting the plants have to remobilize assimilates from present rhizomes which causes a further attenuation of the plant (Szépligeti et al., 2018; Nagy et al., 2019). This shift in mowing regimes that neglected the morphological patterns of plant communities, finally led to an increasing number of monospecific stands of goldenrod, and subsequentely to a rapid decrease in native plant communities. Thus, in 2016 the management authority of Donau-Auen National Park—the Austrian Federal Forests (ÖBf)—started a three-years study in cooperation with the University of Natural Resources and Life Science Vienna.

Of particular interest was the development of a mowing regime, which was not only adjusted on the morphological development of goldenrod but also on the life cycle of native plant communties. In addition, the effectiveness of this mowing regime was tested under different site conditions (dry grassland, humid grassland and nutrient-rich grassland under intensive use). The background of these trials were numerous studies, implying the mowing is the only practiable management options in many conservation areas as (1) it does not represent a serious interference in the soil ecosystem, (2) it can be adapted to site-specific (nature conservation) goals, (3) it can contribute to increase biodiversity and ecosystem services, *i.e.,* prevention of undesireable succession events, temporal adjustment to provide flowerage for wild bees etc. (Smart, Larson & Bauman, 2012; Pál et al., 2015; Szépligeti et al., 2018; Nagy et al., 2021).

An additional goal of the study was to test the efficiacy of other management options. Recently, a couple of studies showed how grazing, flooding or flaming can significantly decrease the abundance of competitive invaders like goldenrod (Le Bagousse-Pinguet, Gross & Straile, 2012; Szépligeti et al., 2017; Nagy et al., 2019; Coughlan et al., 2020). However, in nature conservation areas like the Donau-Auen National Park management options are scarce due to legal regulations, strict planning schemes and restrictions in the management guidelines. Therefore, the only two management options which could be implemented within this study was agricultural cultivation (triticale) and a reverse rotary cutting. Aside the fast aboveground growth of goldenrod, particularly its rhizomes gave a crucial advantage in the fierce competition for water and nutrients. Consequently, grassland mixtures and young trees were regularly prevented from establishment. As shown by Gala-Czekaj, Synowiec & Dabkowska (2021), intensive soil tillage and rotary cutting can effectively contribute in diminishing goldenrod stands by cutting and transferring the rhizoms to the soil surface where they can be negatively affected by different biotic and environmental factors. Thus, both treatments should bring a fast elimination of goldenrod in order to facilitate the establishment of grassland and young trees, respectively.

## Materials & Methods

### Location

The Donau-Auen National Park extends from the north-eastern part of Vienna until the estuary of the river March at the national border of Slovakia. The total area comprises 9.300 ha, consisting of 65% alluvial forest, 20% water areas and 15% managed and unmanaged grassland. The utilization concept of these areas comply to three different zonings (Table 1).

**Table 1:** Zoning of the Donau-Auen National Park.

**Zone**	**Management**
Nature reserve zone	No landuse and no measures which would have impact on environment, landscape or ecological inventory
	Temporary measures are only allowed if they support the natural development
Nature reserve zone with management actions	In general, there are no measures taken, except they support the achievement of nature conservation aims (*i.e.*, mowing of grassland to preserve biodiversity and/or habitats for valuable plant species)
Outer zones	All water ways and canals, touristic areas as well as cultivated areas (field) and buildings like administrative offices or flood protections dams

### Experimental design & measurements

On all trial sites, site-specific transects across the full width/length, consisting of twelve 1 × 1 m permanent trial plots were implemented on each trail site. As all treatments were performed in good agricultural practice throughout the whole area of the sites, we drove 10 × 10 cm metal spikes into the soil which could be easily retrieved with a metal detector during the monitoring. Therefore, an undisturbed mowing and triticale cultivation was secured and an unintentional removing or disturbance of the marking was prevented. The number and the height of the goldenrod shoots were recorded three times a year (one day before each treatment and in the third week of July in each year) to test for the effectiveness of the management actions. In addition, periodic vegetation surveys taking coverage percentages for each species according to Braun-Blanquet (1951) were performed to check if a proper mowing regime can diminish goldenrod, thus promoting the establishment/growth performance of potential competitors.

Weather data was gained on a daily basis from a meteorological station on the experimental farm Groß-Enzersdorf of University of Natural Resources and Life Science Vienna, which is approx. 10–15 km away from the trial sites (48°14′30, 3″N, 16°35′47, 6″E). In the case of goldenrod, the number of growing degree days refers to days with temperatures above 5 °C which were summed up throught the vegetation period (Weber, 2001).

### Treatments

**Figure 1:**
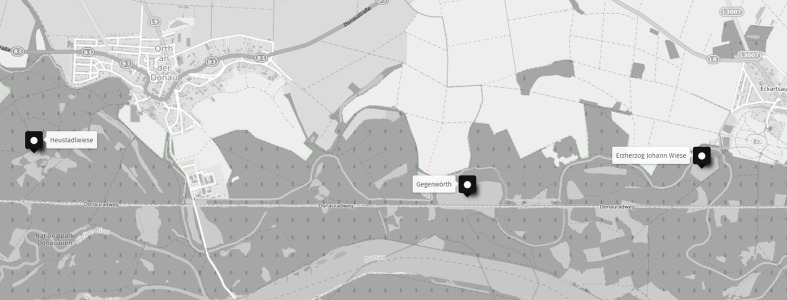
Map of the three trial sites in Donau-Auen National Park.

One prerequisite of this study was that treatments had to be in full accordance with the utilization concept of the national park and the planning schemes for the different sites which were all situated in the “Nature reserve zone with management actions”. Table 1 and Fig. 1 summarize the location of the trial sites as well as the management regimes applied.

#### Mowing

On Heustadlwiese (HW) and Erzherzog Johann Wiese (EJW) a mowing regime with two cuttings per year was implemented. Although the site conditions were completely different, both areas were characterized by a rapidly increasing goldenrod density. HW has a gentle slope from south-east to north-west. In the upper part of the meadow there are dry grassland conditions whereas the lower part of the meadow has properties of a nutrient-rich humid meadow. In contrast, EJW represents an intensively used permanent grassland which is regularly sown to maintain the fodder value as well as a closed turf. A perimeter fence surrounding the grassland area should protect the area from wild animals crossing.

The mowing was performed on the whole area (2.4 ha and 1.5 ha, resp.) with a flail mower (Dragone VP) by staff member of the ÖBf AG. The first cutting took place end of May/beginning of June when goldenrod was in its vegetative stage. The last cutting was executed in late summer/autumn at a time when the dominant grassy species were able to produce and propagate their seeds (Table 2).

**Table 2:** Overview of trial sites, management regimes, and treatment dates 2016–2018.

**Site**	**Coordinates**	**Total area (ha)**	**Treatment**	**2016**	**2017**	**2018**
Heustadlwiese (HW)	48°08′20, 4″N 16°40′53, 3″E	2,4	1st mowing	June, 2	May, 31	May, 22
			2nd mowing	September, 6	August, 31	August, 27
Erzherzog Johann Wiese (EJW)	48°08′13, 9″N 16°47′05, 8″E	1,5	1st mowing	May, 31	May, 30	May, 22
			2nd mowing	September, 1	August, 30	August, 24
Gegenwörth-West (GW)	48°08′05, 3″N 16°44′54, 9″E	5,4	1st Tritcale sowing	November, 9		
			1st Tritcale harvest		July, 10	
			2nd Triticale sowing		October, 10	
			2nd Triticale harvest			July, 4
Gegenwörth-Ost (GO)	48°08′06, 3″N 16°45′13, 1″E	2,5	Reverse Rotary Cutting	November, 11		

#### Triticale sowing

In contrast to HW and EJW, Gegenwörth-West (GW) and Gegenwörth-Ost (GO) represent a typical fallow land after abandonment of agriculture. In 2015, the amended Austrian Agri-Environmental Programme “ÖPUL”, which is part of the European Regional Development Fund (ELER), became effective. Within this subsidy-supported programme, regulations concerning agriculture in national parks were reworked, consequently introducing the obligation to convert a proportional part of agricultural areas into grassland (GW). After the abandondment of the area in 2007, rapidly growing cohorts of goldenrod invaded the area. Efforts to effectively control the stands by seeding grassland mixtures in the years before the trial failed as common management practices which promote the establishment of cultivated grassland like fertilization are not allowed in the national park. As this area was still registered as “agricultural land” we had the possibitly to apply a two years triticale cultivation before grassland seeding.

The idea was, to diminish goldenrod stands by intensive soil tillage followed by a autumn-sown crop which can build up dense, competitive stands before germination or resprouting of goldenrod in spring. This competitive advantage of triticale should force back goldenrod to a minimum, hence to provide a goldenrod-free area for grassland cultivation after these two years of triticale cultivation. Consequently, before sowing, seedbed was prepared with a cultivator and a disc harrow. Due to regular rain events, in 2016 triticale was sown late on 9 November. In 2017, the sowing date was 10 October. In both years, the cultivar Triticano (Saatzucht Probstdorf) was sown in a density of 350 kernels/ha (170 kg/ha) with a seed drill (Pöttinger Vitasem; 4 m operating width). Harvest was performed using a combine harvester (Case Axial-Flow 140; 7.2 m operating width).

#### Reverse rotary cutting

The management plan for GO provided for the conversion of this site into a reforestation area. Therefore, in 2014 over 100 young trees were planted, approx. 70% them seldom woody species like *Prunus* spp. and *Malus* spp. However, due to exessive competition with goldenrod, until the beginning of the trial in 2016 more than half of the young trees died off.

In order to facilitate the establishment and to improve growing conditions for the trees a reverse rotary cutting was executed in autumn 2016 to eliminate the monospecific goldenrod stand on GO in a single work process. Thus, reverse rotary cutting was executed in November 2016 as we hypothesised that the frost during winter will led to freezing off and subsequently to death of these rhizome.

### Statistical analysis

Data analysis was performed using software R, Version 3.4.4 (R Core Team, 2018). For the graphical visualisation of the results we used software SigmaPlot, Version 14.0 (SigmaPlot, 2018). Shannon diversity index (H) was calculated using R package vegan (Oksanen et al., 2018). As the goldenrod shoots on the plots were counted under different time points, a one-way repeated measures analysis of variance was performed using R package nlme, Version 3.1-155 (Pinheiro et al., 2022) with the dependet factor number of shoots and the independet factor year. A Tukey test with a multiple-comparison post hoc Bonferonni correction was perfomed to reveal differences between years, regarding the parametric nature of the analysis, using R package multcomp (Hothorn, Bretz & Westfall, 2008).

## Results

### Weather data

As summarized in Table 3 among years there were severe differences in precipitation sums and the distribution of precipitation during the vegetation periods from March to October. At a total temperature sum of 3,801.5 °C during 2016, 92 days of rain with a precipitation sum of 477.9 mm were recorded. In 2017 the temperature sum of 3.863.6 °C was slightly higher, but there was 33.7% less precipitation which was distributed over 84 days of rain. In particular, 2018 can be classified as a year with extreme weather: With a temperature sum of 4,006.7 °C, only 60 days of rain (precipitation sum: 375.6 mm) were recorded. The comparably high amounts of rain in June and July were all concentrated to 10 heavy rain events.

**Table 3:** Monthly Average temperature (C°) and precipitation sum (mm) in the course of the vegetation period (March–October) in the trial years 2016–2018.

**Year** **2016**	**Av.** **Temp. (C°)**	**Prec.** **(mm)**	**Days of rain (No)**	**Year** **2017**	**Av.** **Temp. (C°)**	**Prec.** **(mm)**	**Days of rain (No)**	**Year** **2018**	**Av.** **Temp. (C°)**	**Prec.** **(mm)**	**Days of rain (No)**
Month				Month				Month			
March	6.4	19.4	10	March	7.4	22.7	6	March	2.4	29.4	10
April	11.0	67.8	13	April	10.5	47.6	12	April	14.7	10.9	4
May	14.7	95.5	12	May	14.5	32.9	14	May	17.7	64.5	6
June	19.7	73.1	17	June	20.7	31.1	7	June	21.2	93.5	10
July	21.7	101.5	14	July	21.7	53.5	16	July	21.5	71.7	11
August	20.9	42.6	7	August	22.4	20.5	7	August	24.1	22.8	8
September	18.5	28.2	4	September	16.5	71.9	11	September	19.0	78.8	8
October	11.3	49.8	15	October	12.5	36.6	11	October	14.3	4.2	3
**Sum**		**477.9**	**92**			**316.8**	**84**			**375.6**	**60**

### Mowing

#### Heustadlwiese (HW)

**Figure 2:**
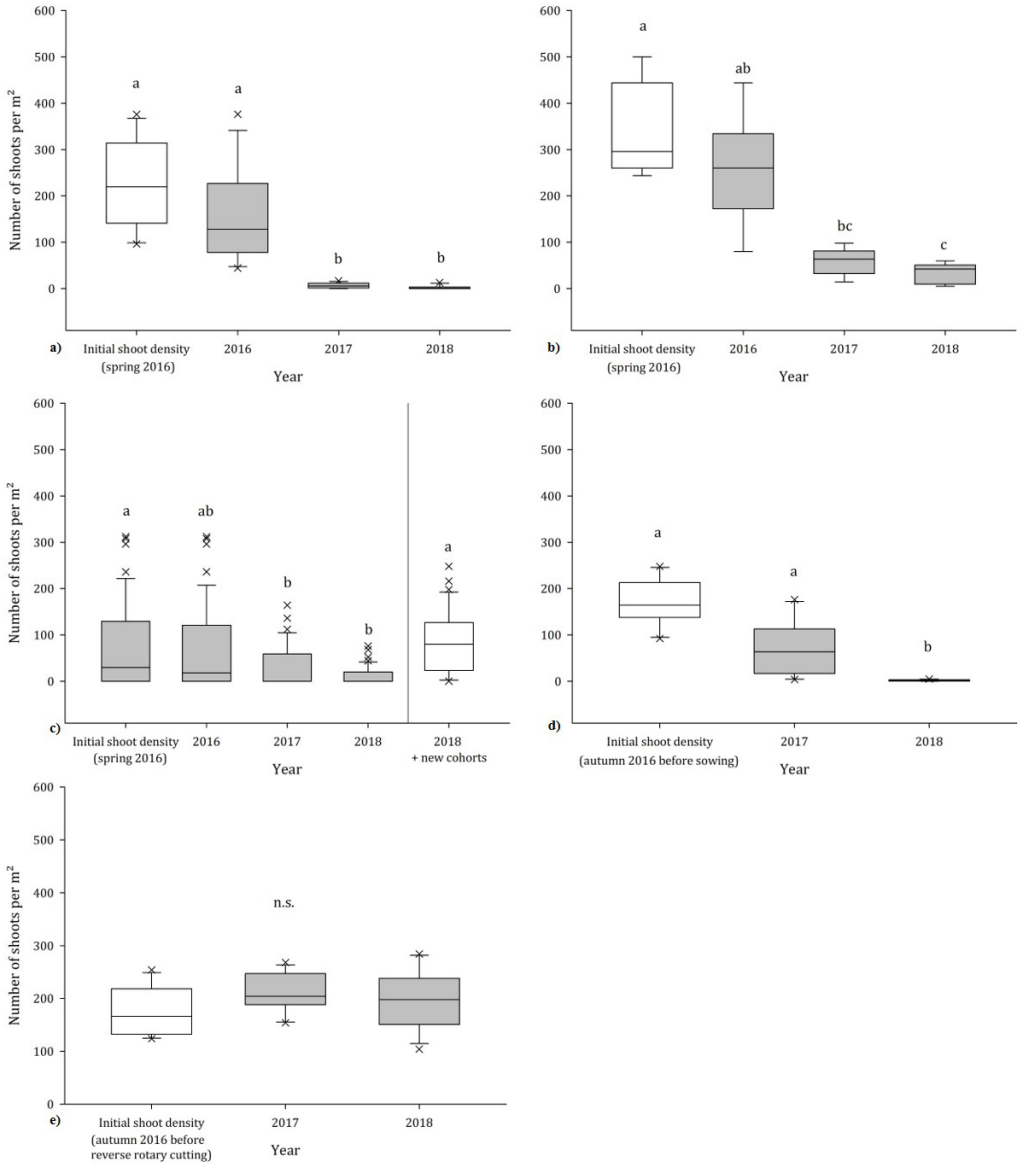
Development of the goldenrod density (2016–2018). Number of goldenrod shoots in dependency of the factor treatment on (A) HW South, (B) HW North, (C) EJW, (D) GW, (E) GO; *n* = 12; different letters indicate significant differences; boxes extend from the 25th to the 75th percentile of each groups distribution of values; vertical extending lines denote adjacent values within 1.5 interquartile range of the 25th and 75th percentile of each group; crosses denote observations outside the range of adjacent values.

The highest decrease in goldenrod density was monitored in the southern, higher located parts of HW (Fig. 2A), which is characterized by dry grassland conditions (*F* = 36.07, *p* < 0.001). With two mowing events per year in the juvenile and resprouting stage of the goldenrod (growth height: 20–25 cm), the shoot density was significantly reduced by 87.9% within two year. As a consequence, in 2017 an increase in native grass species like *Bromus erectus* or *Dactylis glomerata* as well as herbaceous species, typical for dry grassland like *Inula britannica*, *Fragaria viridis* or *Rhinanthus minor* with simultaneous decrease of goldenrod shoots was observed. This trend continued in 2018 leading to an overall decrease of 95.6% of goldenrod shoots on these plots (Table 4). Nevertheless, this repression of goldenrod had no significant impact on the Shannon diversity which only increased slightly from 1.94 to 2.01 (results not shown). A similar development was also observed on the other, more humid and nutrient-rich part of HW (Fig. 2B) even though the density decrease was less pronounced due to the site-specific topography: In the Northern part HW slopes down, showing a depression characterized by higher soil humidity and biomass accumulation, both favouring the growth of goldenrod. Consequently, at the beginning of the trial a highly dominant stand of approx. 365 shoots per m^2^ was recorded, which severely suppressed and inhibited the establishment and growth of other plant species. Nevertheless, with two mowing events per year the average number of shoots per m^2^ could be clearly reduced by 61.0% towards the end of the trial (*F* = 74.17, *p* < 0.001). This reduction of shoots caused the formation of gaps which were rapidly colonized by various *Bromus* spp. as well as herbaceous species like *Cruciata laevipes* or *Erigeron annuus* (Table 4) but had only a slight impact on the Shannon diversity which rose from 1.31 to 1.79.

**Table 4:** Average coverage percentage of the most abundant species on HW, devided into Southern part (dry grassland conditions) and northern part (nutrient-rich humid conditions), recorded on 1 × 1 m plots according to Braun-Blanquet, *n* = 12.

**Species (beginning of the trial 2016)**	**Coverage %**	**Species (end of the trial 2018)**	**Coverage %**
Southern part (dry grassland conditions)			
*Poa pratensis*	62.1 ± 24.6	*Poa pratensis*	66.6 ± 18.2
*Solidago gigantea*	53.3 ± 36.5	*Inula britannica*	52.9 ± 30.3
*Dactylis glomerata*	50.9 ± 31.4	*Dactylis glomerata*	53.6 ± 24.9
*Fragaria viridis*	25.1 ± 30.6	*Linaria vulgaris*	32.0 ± 23.4
*Linaria vulgaris*	21.2 ± 23.4	*Fragaria viridis, Rhinantus minor*	27.6 ± 21.5
Norther part (nutrient-rich humid conditions)			
*Solidago gigantea*	89.9 ± 13.5	*Solidago gigantea*	66.9 ± 12.7
*Urtica dioica*	25.7 ± 22.1	*Urtica diocia*	41.7 ± 16.1
*Poa pratensis*	21.8 ± 22.1	*Poa pratensis*	38.4 ± 18.0
*Cruciata laevipes*	11.8 ± 17.3	*Cruciata laevipes*	26.7 ± 15.4
*Dactylis glomerata*	10.7 ± 11.2	*Galium album*	20.2 ± 10.6

#### Erzherzog Johann Wiese (EJW)

Due to intensive grassland use including regular seeding activity, the site was characterized by typical grassland species like *Poa pratensis*, *Festuca pratensis* or *Trifolium pratense* which effectively suppressed the formation of dominant goldenrod stands (Table 5).

**Table 5:** Average coverage percentage of the most abundant species on EJW, recorded on 1 × 1 m plots according to Braun-Blanquet, *n* = 12.

**Species (beginning of the trial 2016)**	**Coverage %**	**Species (end of the trial 2018)**	**Coverage %**
*Poa pratensis*	63.4 ± 24.4	*Poa pratensis*	59.5 ± 13.4
*Festuca spp.*	58.6 ± 26.7	*Festuca spp.*	50.5 ± 17.2
*Arrhenaterum elatius*	55.3 ± 24.4	*Arrhenaterum elatius*	48.6 ± 18.7
*Trifolium repens*	37.4 ± 30.5	*Solidago gigantea*	43.1 ± 41.3
*Solidago gigantea*	33.5 ± 42.5	*Trifolium repens*	31.3 ± 24.2

**Figure 3:**
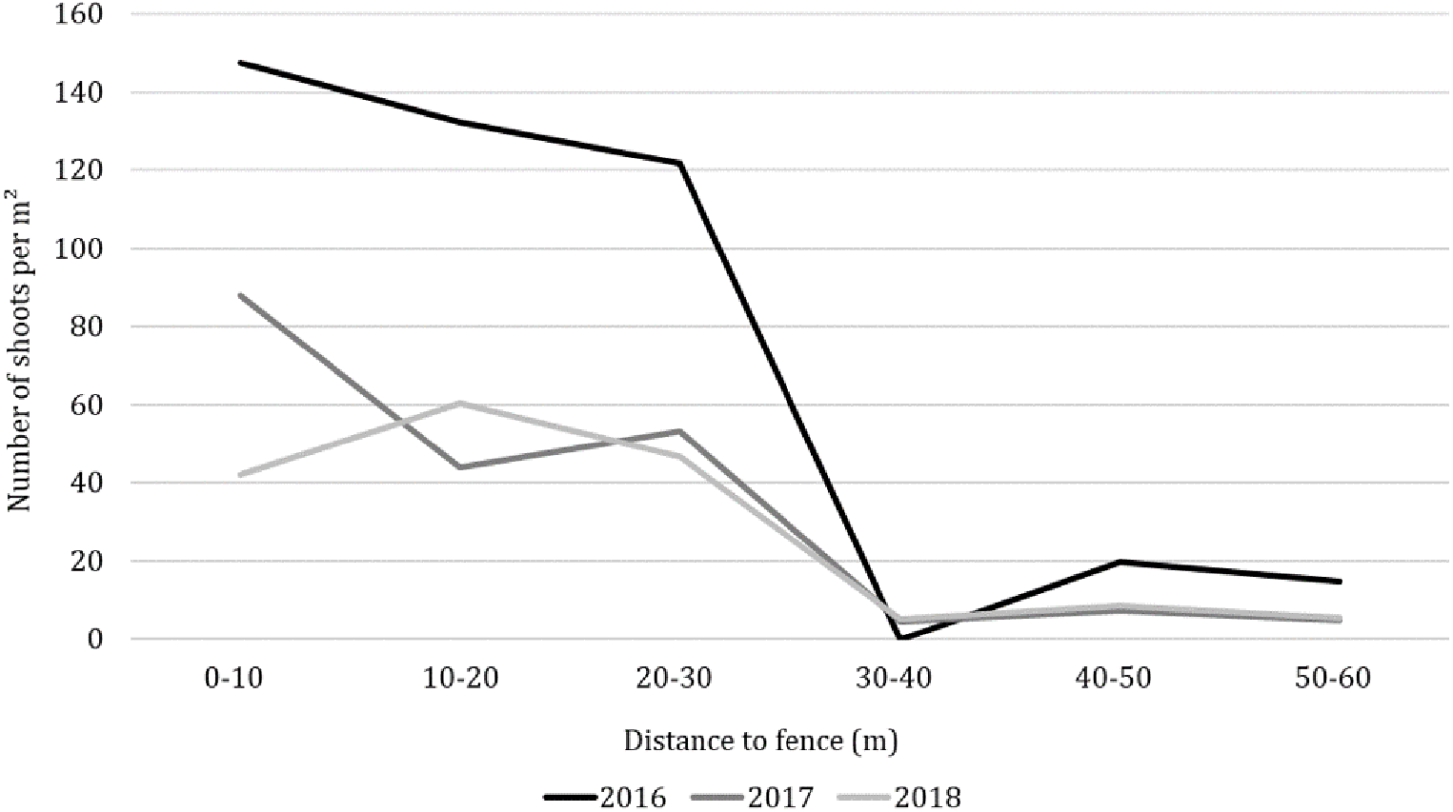
Distribution of goldenrod on site EJW in relation to fence distance.

However, the unmanaged stands at the perimeter fence in the transition area of meadow and forest as well as those within the protection enclosures of the young trees resulted in a steady redelivery of seeds into the soil seed bank. Hence, small gaps in the turf could be enough to ensure sufficient light supply for these seeds to germinate. The high contamination potential of this steady seed input became obvious with increasing distance away from the perimeter fence: At the beginning of the trial, we found on average 172 shoots per m^2^ on the first 30 m distance to the fences. This number was reduced to 27 shoots on the next 30 m. Particularly in the middle of the meadow (trial plots 8–10, Fig. 3) in none of the trial years a goldenrod shoot was counted.

Thus, to avoid bias in the results, after the first counting the goldenrod cohorts within the protection enclosures and on the perimeter fence were removed to prevent further seed propagation into the trial site. Consequently, after one year the goldenrod density within the first 60 m distance to the fence decreased by 40.3%. Until the end of the trial in autumn 2018 a reduction of 68.3% was achieved by two mowing events per year. Over all plots, on average the number of goldenrod shoots decreased significantly from 78.8 per m^2^ in 2016 to 12.2 in 2018 (*F* = 5.91, *p* = 0.002, Fig. 2C).

However, this management success at first glance has to be relativized as in early summer 2018 new goldenrod cohorts popped up in the surrounding of the trial plots due to intensive burrowing of wild boars, which caused in some places huge areas of bare soil. Subsequently, these dug up spots were rapidly invaded by goldenrod (Fig. 2C). As a result, 6 weeks after the burrowing 36 new goldenrod shoots per m^2^ were found on average on twelve dugged up areas which were situated next to the trial plots. If these new cohorts were taken into account, by the end of the trial the goldenrod infestation of EJW even increased by 4.7% compared to the beginning of the trial in spring 2016. This was also depicted by the Shannon diversity index which decreased from 1.88 in 2016 to 1.79 at the end of the trial period.

### Triticale sowing

At the beginning of the trial, we found on average 172 goldenrod shoots per m^2^ with an average ground coverage of 85.8% (Table 6). After triticale cultivation this number could be reduced by 58.5% to 72 shoots within one year.

**Table 6:** Average coverage percentage of the most abundant species on GW, recorded on 1 × 1 m plots according to Braun-Blanquet, *n* = 12.

**Species (beginning of the trial 2016)**	**Coverage %**	**Species (end of the trial 2018)**	**Coverage %**
*Solidago gigantea*	85.8 ± 10.5	*Triticale*	85.3 ± 18.4
*Cirsium arvense*	68.2 ± 26.8	*Setaria verticillata*	22.0 ± 16.7
*Mercurialis annua*	45.2 ± 21.7	*Buglossoides arvensis*	12.6 ± 12.1
*Setaria verticillata*	39.9 ± 16.9	*Veronica spp.*	9.9 ± 13.0
*Asclepias syriaca*	37.1 ± 18.0	*Equisetum arvense*	4.2 ± 8.1

Due to weather- and cultivation problems, in spring the triticale stand was sparse and a high share of plants showed stunted growth, especially in the northern part of the site. Hence, goldenrod density was tightly related to the triticale density: Whereas in areas with nearly no triticale cover 127 shoots per m^2^ were counted, the number decreased to 75 shoots in areas with a triticale cover lower than 50%. In areas with dense triticale cover, on average only nine goldenrod shoot were found. After the second triticale cultivation which took place earlier and under more favourable soil conditions, the average number of goldenrod shoots decreased significantly to 2 per m^2^ (−97.2%, *F* = 43.39, *p* < 0.001) which were all situated in the boundary zone of the site (Fig. 2D).

### Reverse rotary cutting

On the reforestation site, at the beginning of the trial 210 shoots per m^3^ were monitored. The deployment of a reverse rotary cutter did not show any effect on the goldenrod stands. In 2017 we observed only a minimal decrease of 5.4% in goldenrod density (Fig. 2E). The fast developing seedlings together with the sprouting rhizome fragments enabled the goldenrod to build up and establish a monospecific stand again (Table 7).

**Table 7:** Average coverage of the most abundant species on GO, recorded on 1 × 1 m plots according to Braun-Blanquet, *n* = 12.

**Species (beginning of the trial 2016)**	**Coverage %**	**Species (end of the trial 2018)**	**Coverage %**
*Solidago gigantea*	94.6 ± 8.4	*Solidago gigantea*	87.1 ± 13.7
*Poa pratensis*	46.3 ± 34.3	*Cirsium arvense*	58.4 ± 22.7
*Cirsium arvense*	23.5 ± 26.2	*Poa pratensis*	41.1 ± 35.3
*Taraxacum officinalis*	9.2 ± 12.5	*Taraxacum officinalis*	9.3 ± 15.3
*Erigeron annuus*	8.5 ± 12.9	*Erigeron annuus*	6.8 ± 7.9

## Discussion

The results of the study pointed out clearly that the management success of goldenrod is strongly related to the method of control. We examined three different management options (mowing, cultivation and reverse rotary cutting) as they created different types of disturbances not only in the goldenrod stands but also on the location itself. Even though we found that certain treatments were more effective than others, we could not only relate the effectiveness to the intensity of the disturbance created by the control option as particularly weather and site conditions had a strong impact on the management success.

### Mowing

#### Heustadlwiese (HW)

On dry grassland at HW South a mowing regime with two cutting events led to a 95.6% decrease in goldenrod density. In the monospecific goldenrod stand on the more moist and nutrient-rich parts of this site (HW North) this cutting regime caused a goldenrod reduction of only 61.0%. In this context, the initial difference in stem densities between the goldenrod stands on HW South (mean: 226.0 stems/m^2^) and HW North (mean: 341.2 stems/m^2^) could have impact on results. As shown by Smart, Larson & Bauman (2012) and Szépligeti et al. (2018) two mowing events per year are inevitable to achieve substantial management success.

However, both studies indicated that particularly low density stands of goldenrod are more susceptible to intense clipping in late summer than high density clones as the storage of carbohydrate reserves and lateral root growth is significantly reduced.

Even though this three-years mowing treatment had significant negative impact on the goldenrod density, the positive influence on native species presence and diversity was limited. On HW South goldenrod almost disappeared and the newly appearing niches were quickly colonized by already established species like *Poa pratensis, Fragaria viridis* and *Inula britannica*, which all can reproduce vegetatively *via* runners or creeping shoots/roots, respectively.

After three years of mowing treatment, on HW North goldenrod maintained the most dominant species, but in the gaps which occurred after treatment *Urtica dioica* was able to establish in small cohorts. Furthermore, *Poa pratensis* and typical nutrient indicators like *Cruciata laevipes* and *Galium album* increased their abundance but without having any significant impact on Shannon diversity. This observation confirms other studies, indicating that only long-term management options have significant positive impacts on a sustainable management success, community structure and biodiversity (Cox et al., 2007; Nagy et al., 2019). Invasive species like goldenrod can exert negative impacts on native plant communities by changing biotic factors like soil properties and biogeochemical cycles. This disadvantages for native communities can indirectly promote the recovery and re-establishment of the invaders (and other weedy species) after temporal control (Simberloff & Von Holle, 2000; Nagy et al., 2019). Additionally, there could be a lack in the reproductive potential of native species, *i.e.,* because of a shortage of seeds in the soil seed bank due to a high level of displacement of native species.

#### Erzherzog-Johann-Wiese (EJW)

Particularly the stand development of goldenrod on EJW illustrates the problems which may arise when management options are only executed in a short-term perspective. The stand density of goldenrod on EJW was reduced by 84.5% and the abundance of species characteristic for intensive grassland like *Poa pratensis*, *Festuca spp*., and *Trifolium repens* increased at the end of trial. However, an unpredictable wild boar burrowing event in the last trial year caused even an increase in goldenrod infestation on EJW of 4.7% compared to the initial value. The dug up spots provided sufficient light supply, enabling goldenrod seeds from the soil seed bank as well as rhizome fragments to germinate and sprout. This was additionally accelerated by the climatic conditions (Table 3) as heat and drought in early summer 2018 suppressed the germination as well as the growth of native plant species. Bochenek et al. (2016) showed that seeds of goldenrod in contrast do not display symptoms of innate dormancy, and are characterized by a high seed vigor which could be maintained after storage in a wide range of temperatures, in both dry and moist conditions. This is not only an important physiological trait for the formation of a viable seed bank but also a competitive advantage over native species which are more susceptible to environmental conditions (Weber & Jakobs, 2004; Bochenek et al., 2016). Beside goldenrod, other weedy species like *Cirsium arvense* and annual ruderal species like *Conyza canadensis* or *Capsella bursa-pastoris* with low conservation value were able to establish on the dug up spots leading to a decreased Shannon diversity compared to the beginning of the trial in 2016. Similar succession patterns were observed by Ruprecht (2006) and Fenesi et al. (2015) showing that the early stage of vegetation succession on disturbed areas is characterized by a wide array of disturbance-tolerant ruderal-species and fast colonizing generalist species. However, if a competitive invasive species like goldenrod displaces these species it can easily monopolize resources and hinder the establishment of other species due to its quick capacity for vegetative expansion (Catford et al., 2012; Bartha et al., 2014; Fenesi et al., 2015). Even after successful management, a regular monitoring is therefore as imperative as the prevention of further seed introductions. Particularly on EJW, it became obvious that the management success on a certain site is not only a question of management option used but can be heavily influenced by landscape properties like unmanaged fences and hedges which could be an ideal habitat for goldenrod. One of the rare studies dealing with the role of landscape elements in the process of invasion was introduced by Heringer et al. (2019), indicating that landscape elements could (1) represent new established micro-habitats which would not be naturally found on the specific site and (2) lead to more permeability between habitats, enabling species to overcome natural barriers like poor-light forests. Both could be observed at EJW where goldenrod density within a distance of 30 m from the perimeter fence and the protection enclosures was six-times higher than on the other area. Furthermore, the surrounding of these landscape elements represented ideal habitats as the establishment potential of goldenrod was limited by the poor-light surrounding forest as well as the intensive grassland with full soil coverage. Such usually unmanaged stands could be an almost infinite source of seed introduction into the soil seed bank.

### Cultivation and reverse rotary cutting

#### Gegenwörth-West (GW) and Gegenwörth-Ost (GO)

The most intensive methods had fluctuating effects on the goldenrod abundance and had also the smallest positive influence on diversity in these communities. In the first year, triticale cultivation on GW only caused a comparably low reduction of goldenrod by 58.5%. This poor management success could be explained by a combination of site exposition and unfavourable weather condition. The northern part of site is adjacent to a forest and is therefore more shaded and windless. Thus, humidity levels and consequently soil conditions were different to more southern parts of the site which are bright and open. Because of regular rain events until end of October 2016 (Table 3) cultivation had to be postponed again and again until beginning of November when soil conditions allowed a tractor access on the site. However, during sowing the northern part was subject to partly severe soil compactions caused by the tractor tyres as the soil conditions where more wet than in the other parts of the site. In addition, due to the late sowing a considerable number of triticale plants were not able to build up enough biomass before beginning of winter, consequently leading to partly lethal frost damages of the juvenile plants.

After the second triticale cultivation, which took place earlier and under more favourable soil conditions, the average number of goldenrod shoots decreased by −97.2%.

In contrast, reverse rotary cutting on GO had almost no effect on the goldenrod density. The milling caused not only an elimination of all plants (including potential competitors) but also a further fragmentation of the goldenrod rhizome. Because of the mild winter 2017/18 it can be assumed that the rhizomes were not severely affected by frost (Weber & Jakobs, 2004). Additionally, due to the outstanding cold, March 2018 (monthly average: 2.3 °C) germination and juvenile development of indigenous plant species was delayed. Consequently, native species had no initial advantage over the goldenrod. This was exacerbated by the sharp temperature increase in April 2018 (monthly average: 14.7 °C), which aligned the germination time of native plant species and goldenrod. Similar effects with high management intensities were obtained by Nagy et al. (2019). They emphasised that intensive disturbance regimes had negative impact on the already low diversity by consciously removing potential competitors, hence facilitating secondary invasion by goldenrod, as it was the case on GO.

## Conclusions

The results of the study showed clearly that different management can contribute to effectively repress goldenrod stands in a nature conservation area. In particular, a short-term mowing treatment of three years can effectively reduce goldenrod density and simultaneously increase the establishment success of native species. Nevertheless, to improve species richness and community diversity a long-term approach is required as the management success of goldenrod in natural environments can be very sensitive to unpredictable factors like weather and wild animal disturbances. This is particularly true for nature conservation areas where management options are scarce due to legal regulations, strict planning schemes and restrictions in management guidelines. Therefore, we recommend a site-specific management plan which supports the effective reduction in abundance of goldenrod, but also has positive impact on species diversity, which contributes in the enhancement of community resistance against further invasions.

## Supplemental Information

10.7717/peerj.13161/supp-1 Table S1 Number of goldenrod shoots (mean ± standard deviation) throughout the trial period (2016–2018) in dependency of the factor year; results of repeated-measure ANOVA, *n* = 12

10.7717/peerj.13161/supp-2 Data S1 Raw data

## Additional Information and Declarations

### Data Availability

The following information was supplied regarding data availability: The raw data is available in the Supplemental File.
