# Supplementary material for: The management success of the invasive late goldenrod (Solidago gigantea Aiton.) in a nature conservation area is strongly related to site, control measures and environmental factors
Source: PeerJ. 2022 Apr 19;10:e13161. doi: 10.7717/peerj.13161 (PMC9029358; doi:10.7717/peerj.13161)
Supplement: Table S1 [file peerj-10-13161-s001.docx]

| **Site** | **No. of shoots**  **initial density** | **No. of shoots**  **2016** | **No. of shoots**  **2017** | **No. of shoots**  **2018** | **F-value** | **p-value** |
| --- | --- | --- | --- | --- | --- | --- |
| Heustadlwiese (HW dry) | 227.6 ± 95.4 | 153.0 ± 97.7 | 6.8 ± 5.6 | 2.5 ± 4.1 | 36.1 | <0.0001 *** |
| Heustadlwiese (HW humid) | 341.1 ± 96.6 | 253.8 ± 110.7 | 56.6 ± 28.3 | 33.3 ± 20.8 | 74.2 | <0.0001 *** |
| Erzherzog Johann Wiese (EJW)  without digging  with digging | 99.5 ± 91.1 | 65.0 ± 90.1 | 30.3 ± 42.9 | 12.2 ± 19.1  82.4 ± 65.6 | 5.9  2.5 | 0.0024 **  0.0805 ^n.s.^ |
| Gegenwörth-West (GW) | --- | 171.6 ± 48.0 | 71.2 ± 58.2 | 1.8 ± 1.5 | 43.3 | <0.0001 *** |
| Gegenwörth-Ost (GO) | --- | 175.3 ± 44.7 | 209.5 ± 36.4 | 198.1 ± 54.2 | 0.5 | 0.4881 ^n.s.^ |
